# Supplementary material for: MatriCom, a single-cell RNA-sequencing data mining tool to infer cell–extracellular matrix interactions
Source: J Cell Sci. 2025 Jul 11;138(13):jcs263927. doi: 10.1242/jcs.263927 (PMC12276803; doi:10.1242/jcs.263927)
Supplement: Supplementary information [file joces-138-263927-s1.pdf]

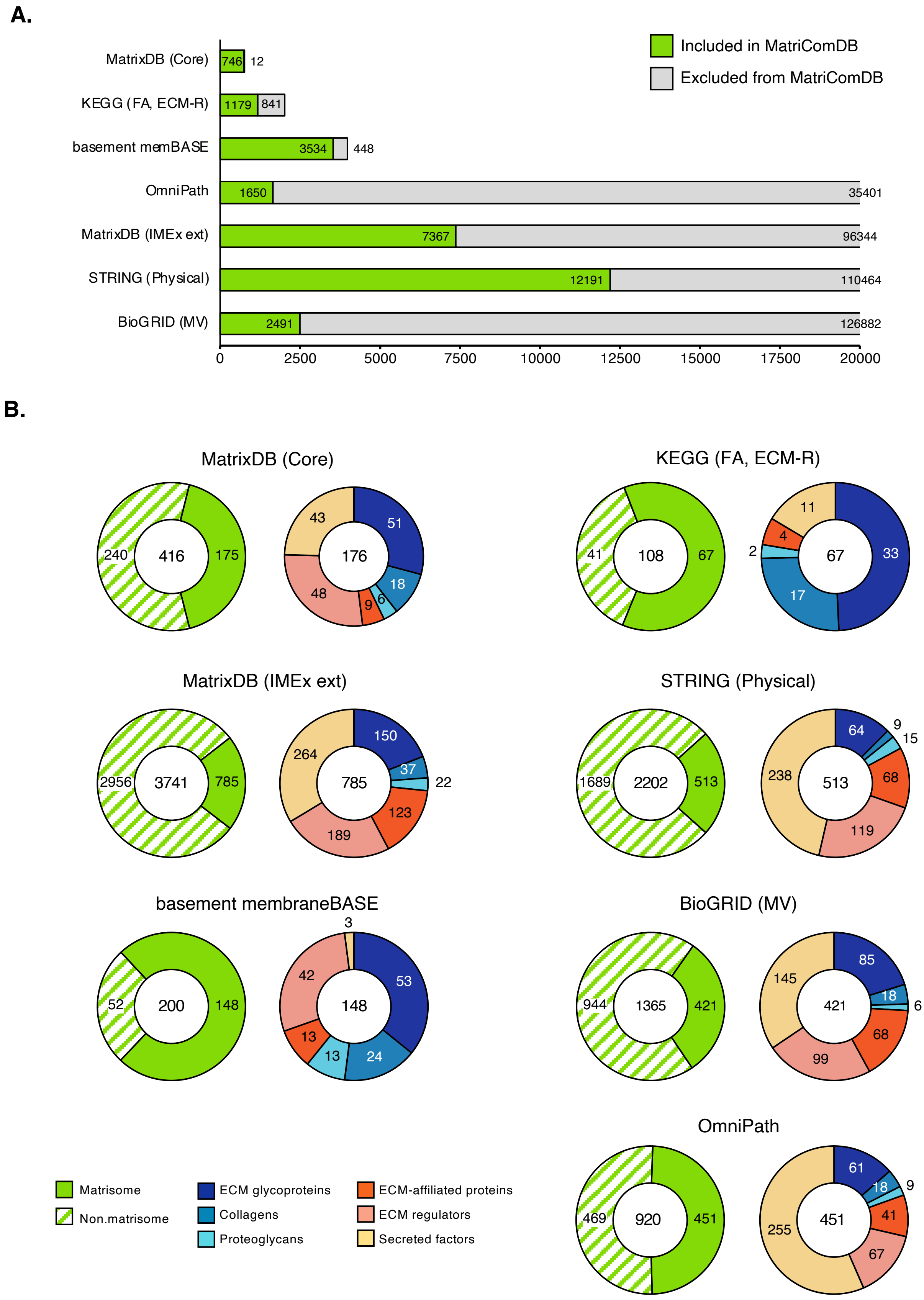

Journal of Cell Science • Supplementary information

Fig. S1. Source databases used to build MatriComDB

(A) Bar chart represents the number of interactions retrieved from each source interaction database and whether these interactions involved at least a matrisome component (green) and thus included in MatriComDB or no matrisome components (grey) and thus excluded from MatriComDB.

(B) Donut charts represent, for each source database, the number of matrisome and non-matrisome components (left panels) involved in interactions compiled in MatriComDB and the proportion of matrisome components across each matrisome category (right panels).

Journal of Cell Science • Supplementary information

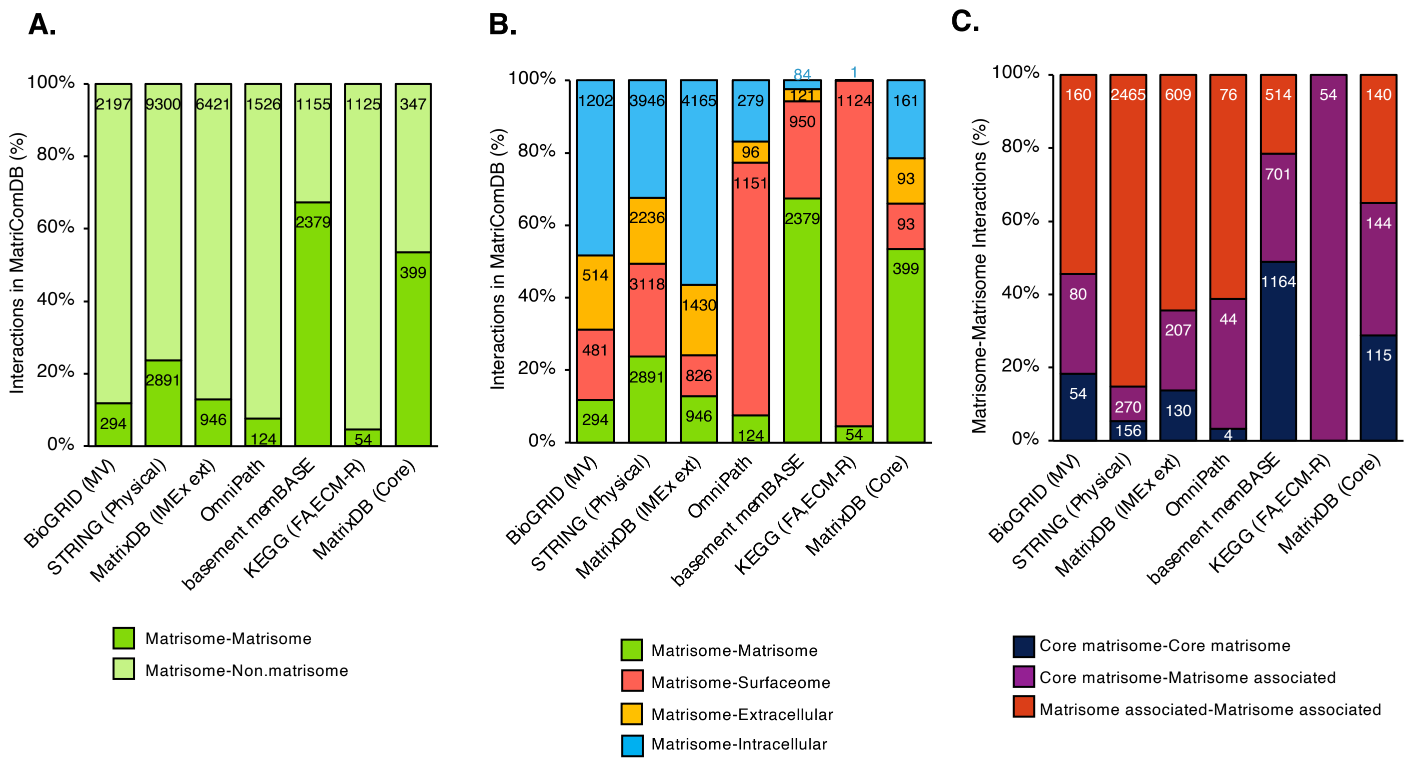

Fig. S2. Contribution of source databases to MatriComDB

(A) Bar chart depicts, for each of the seven interaction databases sourced, the number of interactions included in MatriComDB that involve either one (light green) or two (green) matrisome components.

(B) Bar chart depicts, for each of the seven interaction databases sourced, the number of matrisome protein-matrisome protein (green), matrisome protein-surface protein (red), matrisome protein-extracellular protein (yellow), and matrisome protein-intracellular protein (blue) interactions included in MatriComDB. The assignment of a protein to a subcellular or extracellular compartment is described in the Methods section.

(C) Bar chart depicts, for of the seven interaction databases sourced, the number of interactions between two matrisome proteins listed in MatriComDB involving two core matrisome components (dark blue), one core matrisome component, or one matrisome-associated component (purple), or two matrisome-associated components (red).

A.

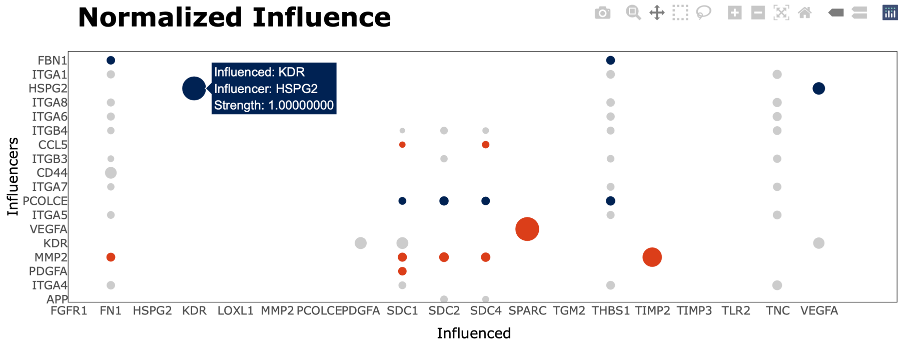

Show 10 entries | Search:

Influencer	Influenced	Strength
COL18A1	CCL2	1
DCN	FGF7	1
FGF1	FGFR1	1
HSPG2	KDR	1
TIMP2	MMP2	0.5

B.

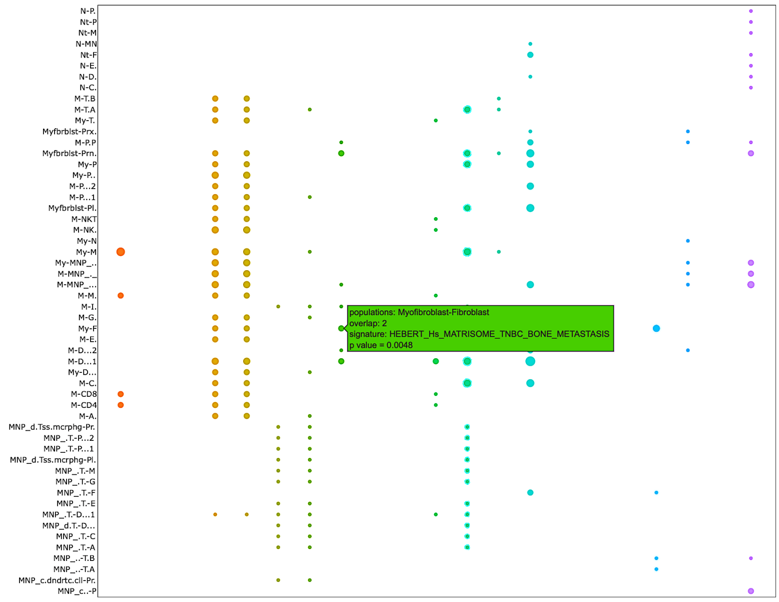

Show 10 entries | Search:

Populations	Signature	Overlap	p.value
Myofibroblast-Fibroblast	HEBERT_Hs_MATRISOME_TNBC_BONE_METASTASIS	2	p value = 0.0048
Myofibroblast-Pelvic.epithelium	DI.MARTINO_Hs_MATRISOME_HIGHLY_PROLIFERATIVE_HNSCC	2	p value = 0.0286
Myofibroblast-Pelvic.epithelium	DI.MARTINO_Hs_MATRISOME_HIGHLY_PROLIFERATIVE_HNSCC_TUMOR_CELL_DERIVED	2	p value = 0.0118
Myofibroblast-Pelvic.epithelium	HEBERT_Hs_MATRISOME_TNBC_LUNG_METASTASIS	2	p value = 0.0211

Journal of Cell Science • Supplementary information

Fig. S3. MatriCom data output

(A) Data tables and bubble charts depicting ‘Network Influencers’ are available through the second tab of the navigation bar on the MatriCom application home page. Hovering over individual bubbles shows data labels. Users can select the number of entries shown per page (black arrows) or query specific terms using the search bar (red arrows). *Exemplary data tables of Network Influencers are provided in Tables S3B and S3E.*

(B) Data tables and bubble charts depicting ‘Enrichment Analysis’ are available through the third tab of the navigation bar on the MatriCom application home page. Hovering over individual bubbles shows data labels. Users can select the number of entries shown per page (black arrows) or query specific terms using the search bar (red arrows). *Exemplary data tables of the Enrichment Analysis are provided in Tables S3C and S3F.*

Journal of Cell Science • Supplementary information

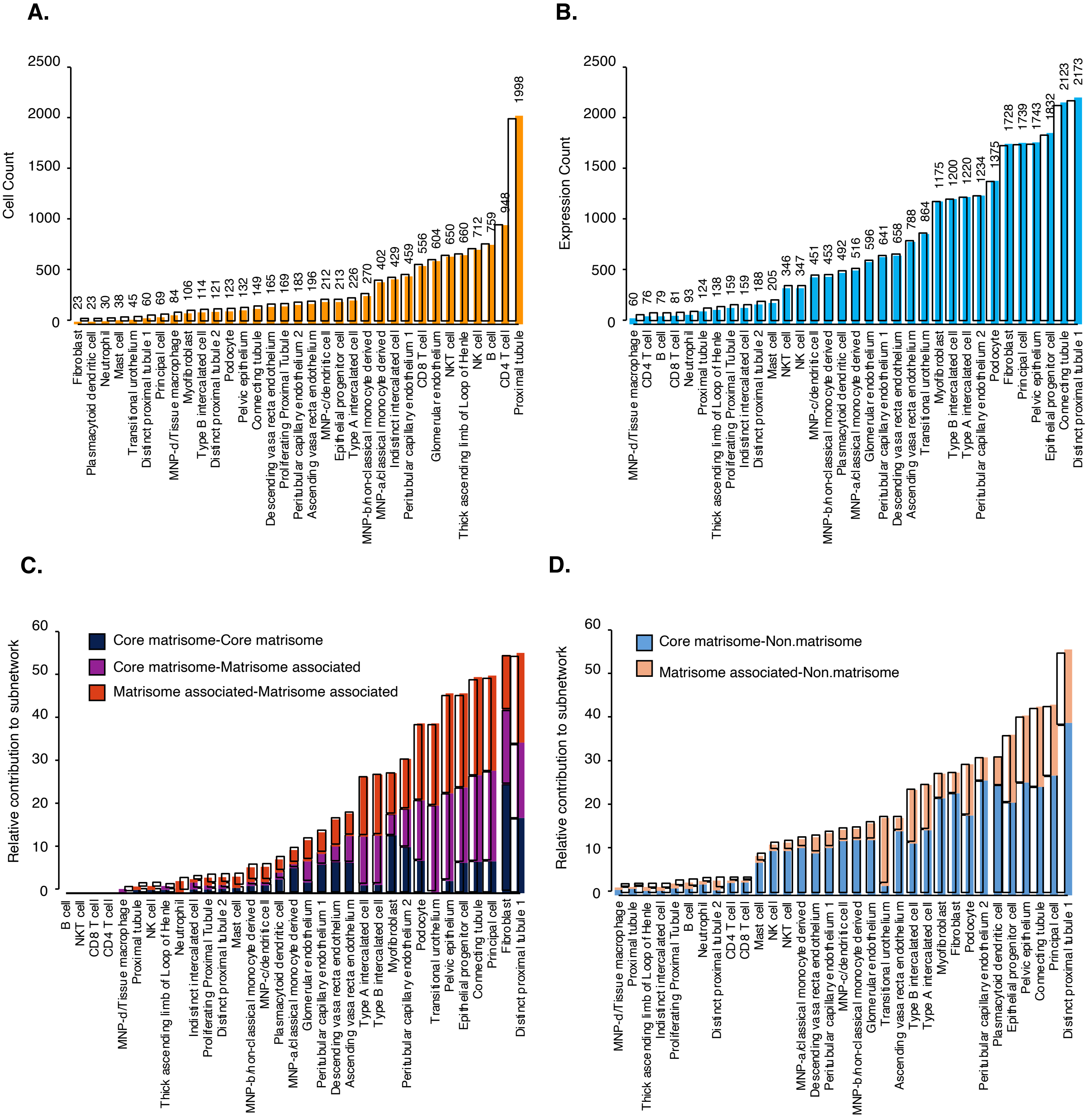

Journal of Cell Science • Supplementary information

Fig. S4. Matrisome communication network of the kidney

(A) Bar chart represents the number of cells per population in the open-access kidney scRNA-Seq dataset in the MatriCom output, see also Table S5.

(B) Bar chart represents the number of instances each population appears in any communication in the MatriCom output, see also Table S5.

(C) Bar chart represents the relative contribution of each cell population partnering with fibroblasts to the matrisome-matrisome communication network and the core matrisome-core matrisome (dark blue), core matrisome-matrisome associated (purple), or matrisome associated-matrisome associated (red) subnetworks.

(D) Bar chart represents the relative contribution of each cell population partnering with fibroblasts to the core matrisome-non.matrisome (light blue) and matrisome associated-non.matrisome (salmon) communication network.

Journal of Cell Science • Supplementary information

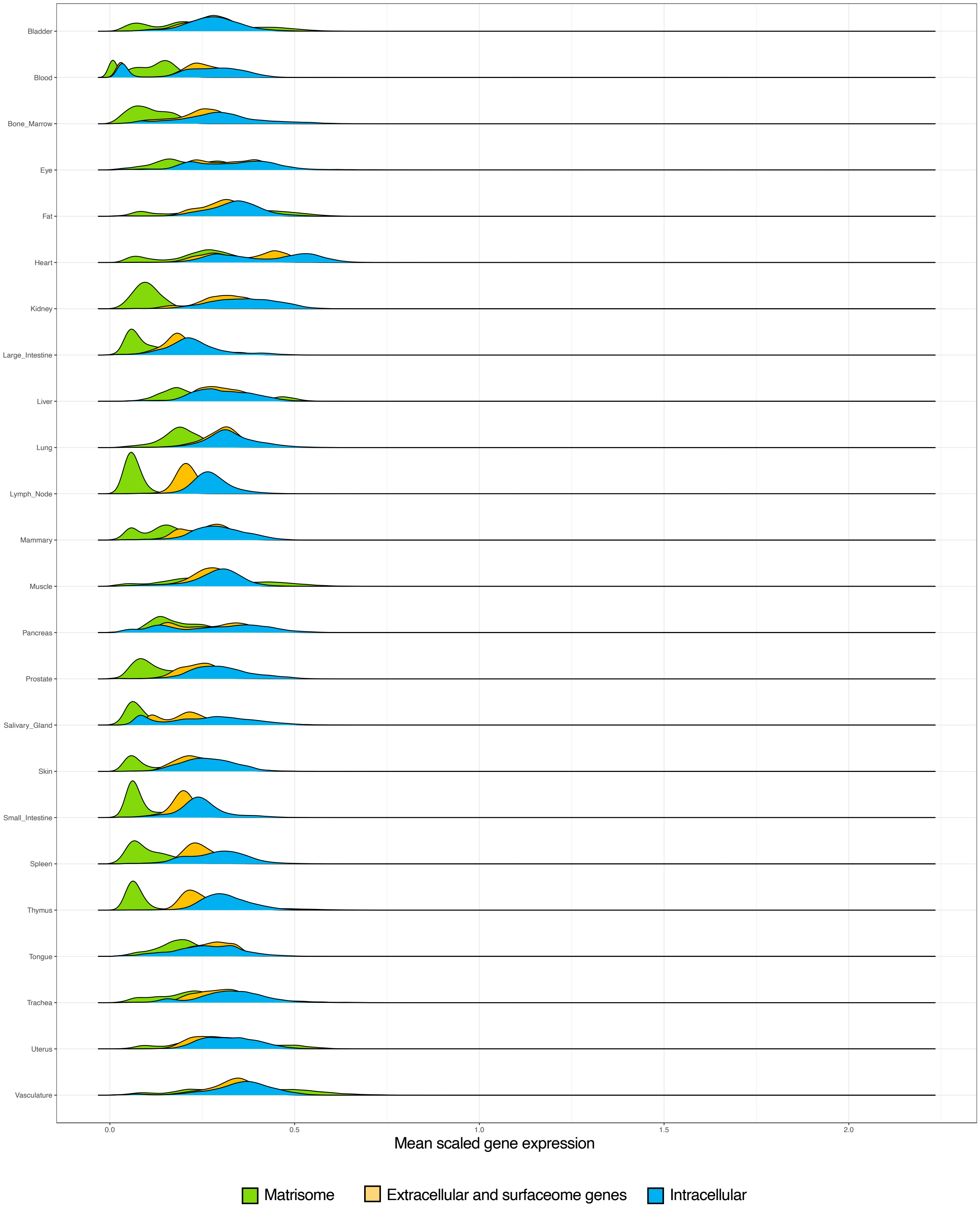

Journal of Cell Science • Supplementary information

Fig. S5. Distribution of gene expression levels across the 24 organs and tissue of the Tabula Sapiens dataset

Distribution of mean normalized gene expression levels for matrisome, surfaceome/ extracellular (non-matrisome), and intracellular genes in Tabula Sapiens. *** $p < 0.001$, one-way ANOVA.

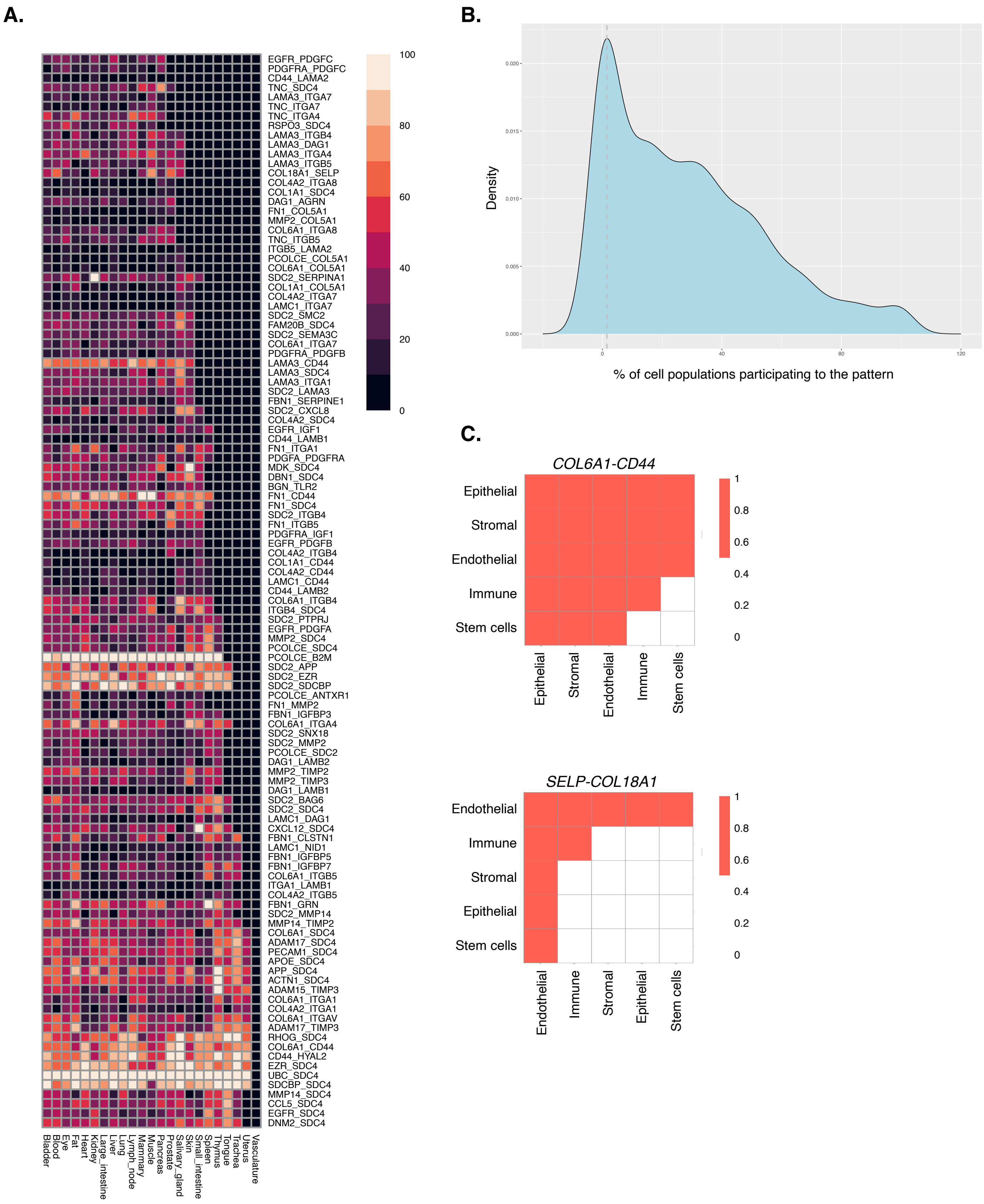

Journal of Cell Science • Supplementary information

Fig. S6. Pleiotropy and specialization of matrisome communication patterns

(A) Heat map represents the percentage of cell populations participating in each pattern per organ.

(B) Density distribution of % cell populations per pattern. Note the first mode at approx. 1.5% (dotted gray line).

(C) Examples of pleiotropy and specificity of the patterns. Some communication pairs (*e.g.*, *COL6A1-CD44*) are highly pleiotropic and used by cells of the stromal, epithelial, endothelial, and immune compartments across different organs, while other pairs (e.g., *SELP-COL18A1*) are highly specific and mediate almost exclusively interactions with the endothelial compartment.

Journal of Cell Science • Supplementary information

A.

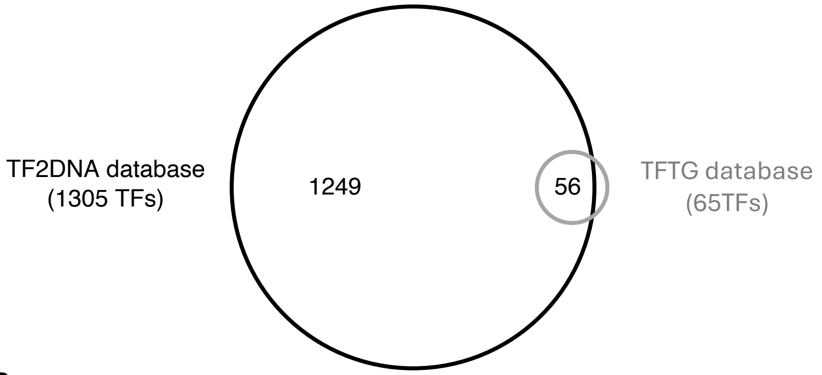

B.

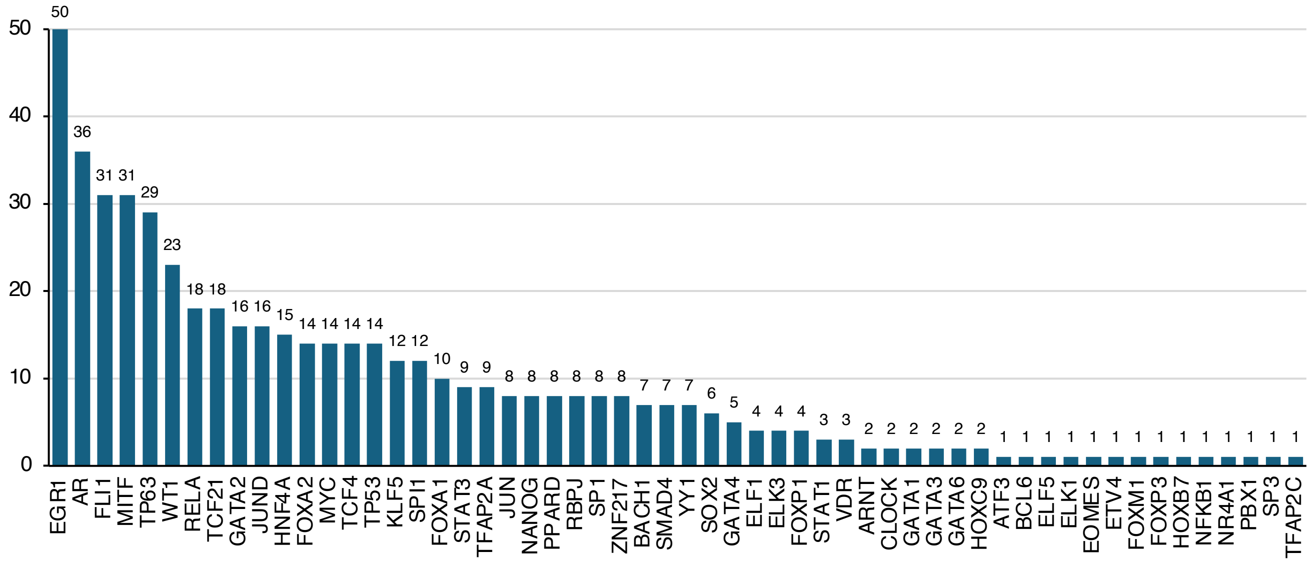

C.

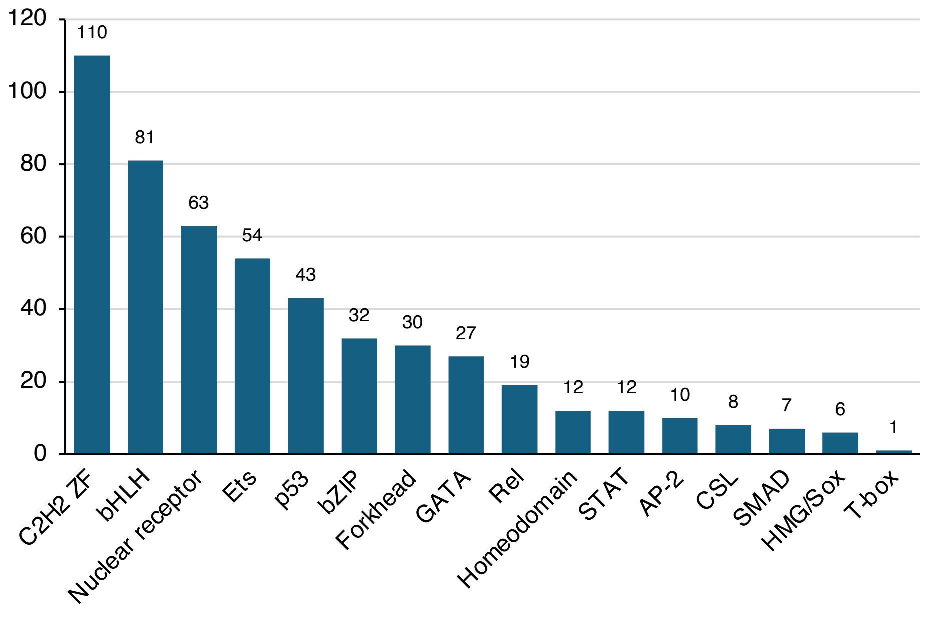

Journal of Cell Science • Supplementary information

D.

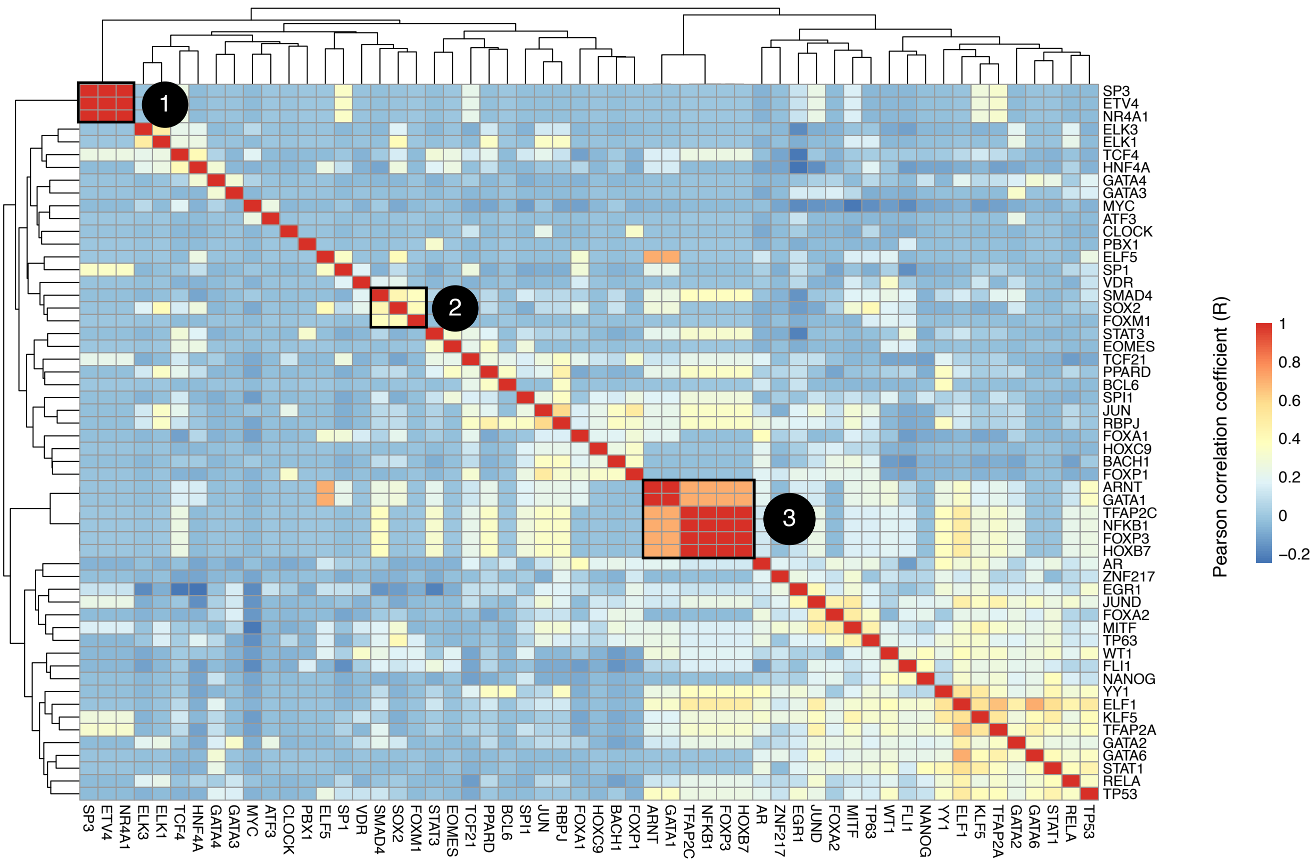

Fig. S7. Transcriptional regulation of matrisome communication pairs

(A) Venn diagram represents the number of transcription factors (TFs) with reported ability to regulate both genes of the matrisome communication pairs identified above were mined from two independent databases (TF2DNA and TFTG).

(B) Bar chart represents the number of matrisome communication pairs regulated by the 56 TFs found in both the TF2DNA and the TFTG databases.

(C) TFs were grouped by family. Bar chart represents the number of matrisome communication pairs regulated by TF families.

(**D)** Heat map represents the correlation between TFs regulating matrisome communication pairs, based on a binary responsibility matrix where each TF is marked as "1" if regulating a pair and "0" otherwise. Correlation analysis identified patterns of similarity, or clusters. Exemplary clusters were identified by visual inspection and greedy modularity optimization of the correlation matrix after thresholding for strong correlation (Pearson R > 0.7) only.

Journal of Cell Science • Supplementary information

Table S1. Contribution of different source databases to MatriComDB

(A) Table reports the total number of interactions retrieved from each source database and the number of interactions excluded (column C) or included (column D) in MatriComDB. MatriComDB interaction counts are further classified by cellular compartments and matrisome divisions of the communicating proteins they involve (columns E-L).

(B) Table reports the number of unique genes from each source database represented in MatriComDB (columns B-D). Matrisome genes are further broken down by matrisome categories (columns E-J).

Available for download at
https://journals.biologists.com/jcs/article-lookup/doi/10.1242/jcs.263927#supplementary-data

Table S2. List of open-access collections and datasets included in MatriCom

The MatriCom sample dataset repository consists of 59 individual scRNA-Seq datasets from three open-access collections: Tabula Sapiens (**S2A**), The Human Protein Atlas (**S2B**), and additional open-access datasets (**S3C**). This table provides information, including sample metadata and links to original references, database entries, and downloadable data files, for each sample scRNA-Seq dataset.

Available for download at
https://journals.biologists.com/jcs/article-lookup/doi/10.1242/jcs.263927#supplementary-data

Table S3. Output from MatriCom analysis of a kidney OA sc-RNA-Seq dataset with original and Census cell annotations

This table provides examples of the tabular data generated upon running MatriCom on the open-access kidney sample dataset using default query parameters and filter settings. Data represents the raw files downloaded using the ‘Export tabular data (XLSX)’ button.

(A) ‘Communication network’ table with original cell annotations

(B) ‘Network influencers’ with original cell annotations

(C) ‘Enrichment analysis’ table with original cell annotations

(D) ‘Communication Network’ table with Census cell annotations

(E) ‘Network influencers’ table with Census cell annotations

(F) ‘Enrichment analysis’ table with Census cell annotations

Available for download at
https://journals.biologists.com/jcs/article-lookup/doi/10.1242/jcs.263927#supplementary-data

Table S4. Matrisome communication network of kidney sample dataset

This table summarizes the cell- and gene-level matrisome communication networks identified in the open-access kidney scRNA-Seq dataset (https://www.ebi.ac.uk/biostudies/studies/S-SUBS7).

(A) Relative gene-level contribution of individual populations to the kidney matrisome communication network.

(B) List of communications in the fibroblast-specific subset of the kidney matrisome communication network defined as any communication involving a gene expressed by fibroblasts.

(C) Relative communication-level contribution of partner populations to the fibroblast-specific communication network, classified by communication type.

(D) Relative contribution of partner populations to the matrisome-matrisome subset of the fibroblast communication network, broken down by the classification of communicating genes by matrisome division.

(E) Relative contribution of partner populations to the matrisome-non.matrisome subset of the fibroblast communication network, broken down by matrisome division of the communicating genes.

Available for download at
https://journals.biologists.com/jcs/article-lookup/doi/10.1242/jcs.263927#supplementary-data

Journal of Cell Science • Supplementary information

Table S5. ECM/ECM-receptor communications within the fibroblast subset of the kidney matrisome communication network

This table summarizes the distribution of fibroblast ECM-receptor communications reported in the kidney matrisome communication network according to the relative contribution of receiver populations and matrisome coverage of the ligand-receptor gene pair.

(A) List of fibroblast ECM-receptor communications in the kidney matrisome communication network, defined as any communication represented in the KEGG ECM-receptor interaction (hsa04512) dataset where fibroblasts are the sender population (*i.e.*, express the genes encoding ligands).

(B) List of genes encoding ligands and receptors reported in the fibroblast ECM-receptor communication network.

(C) Relative contribution of receiver populations to the fibroblast ECM-receptor communication network, broken down by matrisome division of the ligand and receptor genes. See also Fig. 4C.

(D) List of fibroblast collagen VI-receptor interactions inferred from communication pairs of the kidney matrisome communication network. Communications were defined as any fibroblast ECM-receptor communication with COL6A1, COL6A2, or COL6A3 as ligands.

(E) List of collagen VI-integrin interactions identified by MatriCom communication network analysis of the sample kidney scRNA_Seq dataset.

(F) List of collagen VI-cell surface receptor (non-integrin) interactions identified by MatriCom communication network analysis of the sample kidney scRNA_Seq dataset.

Available for download at
https://journals.biologists.com/jcs/article-lookup/doi/10.1242/jcs.263927#supplementary-data

Journal of Cell Science • Supplementary information

Table S6. Mapping of The Human Protein Atlas dataset to match Tabula Sapiens tissues for Census cell annotations

This table maps organs and tissues of The Human Protein Atlas datasets to Tabula Sapiens ones, a step required prior to reannotating populations of THPA datasets using Census.

Available for download at
https://journals.biologists.com/jcs/article-lookup/doi/10.1242/jcs.263927#supplementary-data

Table S7. Conserved matrisome patterns across a collection of open-access scRNA-Seq datasets

This table lists the matrisome patterns identified in open-access datasets from Tabula Sapiens and The Human Protein Atlas.

(A) List of the 113 matrisome patterns identified annotated with matrisome divisions and categories.

(B) Number of connections mediated by the patterns across tissue compartments in Tabula Sapiens.

(C) Number of connections mediated by the patterns across tissue compartments in The Human Protein Atlas.

Available for download at
https://journals.biologists.com/jcs/article-lookup/doi/10.1242/jcs.263927#supplementary-data

Table S8. Transcription factors regulating the matrisome patterns

This table lists the transcription factors potentially regulating the co-transcription of partnering genes of matrisome communication pairs enriched with transcription factor families.

Available for download at
https://journals.biologists.com/jcs/article-lookup/doi/10.1242/jcs.263927#supplementary-data

Journal of Cell Science • Supplementary information
